# Emerging flexible and wearable physical sensing platforms for healthcare and biomedical applications

**DOI:** 10.1038/micronano.2016.43

**Published:** 2016-09-26

**Authors:** Kenry, Joo Chuan Yeo, Chwee Teck Lim

**Affiliations:** 1 NUS Graduate School for Integrative Sciences and Engineering, National University of Singapore, Singapore 117456, Singapore; 2 Centre for Advanced 2D Materials and Graphene Research Centre, National University of Singapore, Singapore 117543, Singapore; 3 Department of Biomedical Engineering, National University of Singapore, Singapore 117576, Singapore; 4 Mechanobiology Institute, National University of Singapore, Singapore 117411, Singapore

**Keywords:** Electronic skins, Flexible sensors, Health monitoring, Liquid-state devices, Microfluidics, Tactile sensing

## Abstract

There are now numerous emerging flexible and wearable sensing technologies that can perform a myriad of physical and physiological measurements. Rapid advances in developing and implementing such sensors in the last several years have demonstrated the growing significance and potential utility of this unique class of sensing platforms. Applications include wearable consumer electronics, soft robotics, medical prosthetics, electronic skin, and health monitoring. In this review, we provide a state-of-the-art overview of the emerging flexible and wearable sensing platforms for healthcare and biomedical applications. We first introduce the selection of flexible and stretchable materials and the fabrication of sensors based on these materials. We then compare the different solid-state and liquid-state physical sensing platforms and examine the mechanical deformation-based working mechanisms of these sensors. We also highlight some of the exciting applications of flexible and wearable physical sensors in emerging healthcare and biomedical applications, in particular for artificial electronic skins, physiological health monitoring and assessment, and therapeutic and drug delivery. Finally, we conclude this review by offering some insight into the challenges and opportunities facing this field.

## Introduction

**Figure 1:**
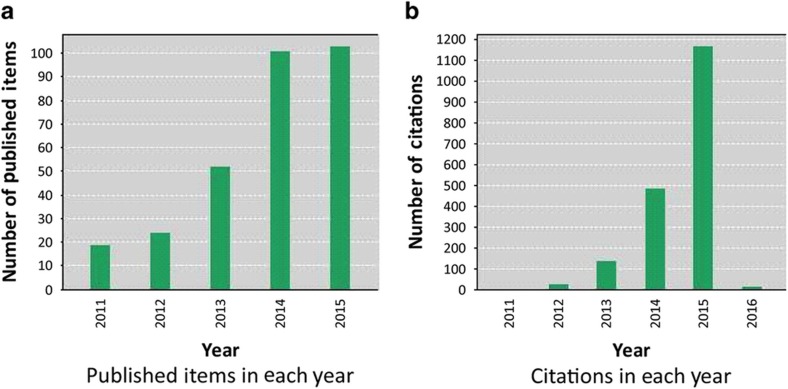
Total number of publications and citations on the topic ‘Flexible and Wearable Sensors’ in the last 5 years. Total number of (**a**) publications and (**b**) citations on the topic of ‘Flexible and Wearable Sensors’ showing a progressive increasing trend from 2011 to 2015. Source: Web of Science, January 2016.

Physical sensing platforms that detect and monitor the surroundings and communicate with the acquired physical data, such as pressure, shear, strain, torsion, temperature, and humidity, form the fundamental building blocks of a multitude of advanced applications, including wearable consumer electronics^1–3^, soft robotics^4–6^, smart medical prosthetics and electronic skins^7–9^, and real-time healthcare monitoring^10,11^. With increasing demand for these applications, there is a corresponding increase in the requirements and criteria for the development and effective implementation of such sensors. As such, recent years have seen the advent of a particular class of physical sensing devices, that is, lightweight, flexible, and wearable physical sensors with distinct functionalities, notably with high degrees of deformability and conformability, long-term stability, increased sensitivity, and excellent optical transparency^12–15^. In general, these sensing devices are functionally optimized for particular platforms and applications and may be stand-alone, portable, wearable, or implantable^10,16^. Compelling evidence of the rapid development of flexible and wearable physical sensing platforms can be traced from the progressive increase in the total number of scientific publications specific to this field over the past several years. Figure 1 shows the total number of scientific publications and citations in the last 5 years obtained through Web of Science based on the keywords ‘Flexible and Wearable Sensors’.

Physical sensors have been designed and fabricated from a plethora of materials, notably flexible materials and substrates^3,17,18^. Flexible materials provide an excellent degree of deformability and conformability on surfaces with various topologies and geometries^19^. This unique characteristic renders them attractive base materials for physical sensors. Normally, flexible sensors are made of substrates such as polycarbonate (PC) and polyethylene terephthalate (PET)^20^, which offer superior deformability and high optical transparency. Nevertheless, another class of flexible substrates, that is, soft silicone elastomers, such as polydimethylsiloxane (PDMS) and silicone rubbers^21^, is of great interest owing to providing additional advantages such as stretchability and compliancy^2^.

Generally, physical sensing platforms operate based on relative variations in their electrical parameters, such as piezoelectricity^22–24^, triboelectricity^25,26^, capacitance^27–30^, or resistance^31–33^, to detect and quantify the desired physical data, including pressure and temperature. Depending on the types of active sensing elements experiencing these changes, these sensors may be largely classified into solid-state and liquid-state sensing devices. As the name suggests, the active sensing element of the solid-state sensor is typically in solid form^3,29–32^. Some examples include nanomaterials of polymers, carbon, semiconductors, and metals, for instance, carbon nanotubes (CNTs)^29–31,34,35^, semiconductor and metallic nanowires^3,36,37^, polymer nanofibers^23,24,32^, and metallic nanoparticles^38–40^. In contrast, physical sensors employing liquid active sensing components, such as ionic^41,42^ and metallic liquids^43^, are classified as liquid-state sensors^41–43^. Irrespective of the type of the active sensing element of the sensors, changes in the electrical parameters of a majority of the physical sensing platforms are induced by the mechanical deformations experienced directly by the sensing elements or imposed by the encapsulating assembly on the sensing components under an applied load. Interestingly, through this simple and effective operating mechanism, an assortment of flexible and stretchable functional sensors have been developed and optimized in recent years for various healthcare and medical engineering applications, notably for artificial electronic skins^7,8,44–46^, physiological monitoring and assessment systems^10,47^, and therapeutic and drug delivery platforms^48–50^.

**Figure 2:**
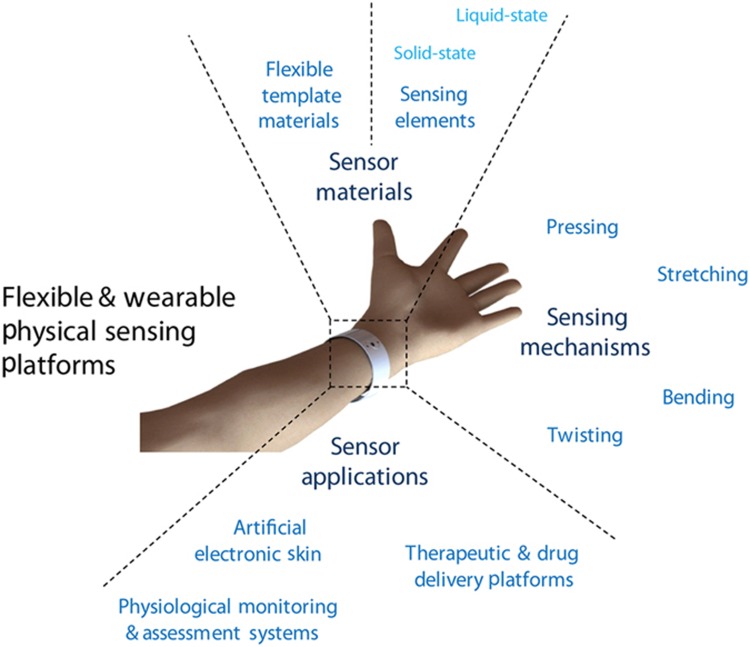
Emerging flexible and wearable physical sensing platforms for healthcare and biomedical applications. Flexible physical sensors comprise two distinct building blocks, that is, the flexible template materials and the active sensing elements, which may take either solid or liquid form. The fundamental sensing mechanism of the flexible and wearable sensors is based on the mechanical deformations experienced by the sensing devices, such as pressing, stretching, bending, and twisting. Emerging applications of flexible and wearable sensors in the healthcare and biomedical fields include artificial electronic skins, physiological monitoring and assessment systems, and therapeutic and drug delivery platforms.

In this review, we offer a broad overview of the flexible and wearable physical sensing platforms emerging for healthcare and biomedical applications in the last 5 years (Figure 2). First, we introduce some examples of commonly utilized flexible materials as well as the fabrication of these flexible and stretchable sensors. Next, we discuss current and emerging physical sensing platforms based on their active sensing components, solid-state and liquid-state physical sensing platforms, and then highlight the deformation-based operating principles of the flexible and stretchable sensors. Subsequently, we looked at the healthcare and biomedical applications of flexible and wearable physical sensors, specifically for artificial electronic skin, *in situ* physiological monitoring and assessment, and therapeutic and drug delivery. Finally, we offer insight regarding the challenges and opportunities facing the development and application of flexible and wearable physical sensing platforms.

## Flexible and stretchable sensor materials and fabrication

### Sensor materials

Flexible and wearable sensors are expected to provide accurate and reliable sensing without compromising the natural movements and comfort of the users. Thus, an important characteristic defining flexible sensors is their skin-like conformability and stretchability. To achieve these properties, flexible thermoplastic polymers, such as polyethylene terephthalate (PET)^51–53^, PC^53^, and polyurethane (PU)^54,55^, have been selected for the fabrication of flexible materials owing to their outstanding optical transparency, ease of fabrication, and superior deformability. In addition to these materials, another type of flexible substrates that has been of great interest recently is the class of soft silicone elastomers, in particular, PDMS^53^ and the trademarked silicone rubbers such as EcoFlex® (Smooth-On, Macungie, PA, USA)^53^, DragonSkin® (Smooth-On, Macungie, PA, USA)^56^, and Silbione® (Bluestar Silicones, East Brunswick, NJ, USA)^56–58^. This group of flexible elastomers provides a high degree of deformability and conformability on different surfaces with varied textures and geometries, rendering them viable candidates for use as one of the fundamental components of stretchable and wearable sensing devices. Furthermore, these flexible silicone elastomers are generally chemically inert and biocompatible, making them excellent for use in implantable flexible sensors^59,60^. Table 1 (A) summarizes the polymeric substrates and soft elastomeric materials typically employed for the fabrication of the soft templates of flexible and stretchable physical sensing devices.

In addition to the soft substrate-based templates, completely functional flexible and wearable sensors require their most important components, that is, the active sensing elements. Although the detection schemes of physical sensors vary widely from mechanical to optical principles^61^, the electrical detection scheme is widely adopted owing to its high sensitivity and reliability. Therefore, commercially available sensors often use solid-state devices, such as piezoelectric, capacitive, and resistive materials. However, materials produced based on conventional electronic technology generally are not flexible and stretchable. Thus, more advanced materials are continually being explored as sensing elements for soft sensors.

Among these advanced materials, inorganic materials in the form of nanofibers or nanowires are being extensively investigated as alternatives owing to their sensitivity and ease of assembly on unconventional materials^3,32,37,61^. Carbon-based nanomaterials, for example, offer superior sensitivity owing to their remarkable electrical and mechanical properties^29,62–65^. These nanomaterials are increasingly being utilized for various sensing applications, including biomolecules^66^, chemicals^61,67,68^, mechanical strain^46,69,70^, and pressure^71^. More recently, 2D nanomaterials such as graphene are also being explored for this purpose. The tunability of the physical properties of these nanomaterials is one of the promising features for their implementation as versatile sensing platforms^72,73^. Indeed, graphene-based physical sensors have been demonstrated in many applications in recent years, including the detection and monitoring of humidity^74^, pH^75^, chemicals^76,77^, biomolecules^73,78–80^, and mechanical forces^81–83^. Moreover, the biocompatibility of graphene opens up further possibilities for its use as a fundamental element of implantable biophysical sensors.

Apart from carbon- and graphene-based nanomaterials, other active sensing components for flexible and wearable physical sensors are also being actively explored and reported. These materials include polymers^23,24,32^, semiconductors^36^, and metallic conductor-based nanomaterials^37–40^ as well as ionic^41,42,84^ and metallic liquids^43,85^. Table 1(B) further provides some of the materials commonly utilized as active sensing elements of flexible sensors with their quantitative specifications. Further progress towards conformal sensors with tunable properties and functionalities for different sensing applications is confidently anticipated, based on the variety of soft flexible templates and active sensing components.

### Sensor fabrication

The development of flexible and wearable sensors demands innovations in both the materials science and fabrication process. Conventionally, sensor fabrication relies on the standard photolithography process. However, this process is largely incompatible with flexible substrates. Therefore, different fabrication routes are required for the production of flexible sensing platforms. Among such approaches, functional printing techniques are the most prevalent manufacturing method. Indeed, printed electronics present an attractive alternative for the patterning of insulative and piezoelectric polymeric substrates, providing the excellent electrical conductivity necessary to become the fundamental building block of functional devices.

In general, the basic ingredient of printed electronics is conductive ink, which typically contains a liquid suspension of metallic particles or inorganic materials. This unique composition allows the uniform deposition of the conductive ink on the desired polymeric substrates and subsequent curing of the ink-coated substrates at high temperatures. Unfortunately, owing to the high sintering temperature requirement, this technique is initially restricted to a small number of thermoplastics such as polyimide (PI)^53,86^. Nevertheless, advances in material synthesis and device fabrication have since mitigated the high temperature requirement such that more affordable thermoplastic alternatives, such as polyethylene naphthalate (PEN)^52,53,87^, may be used for this purpose as well.

Conductive ink printing may be performed based on a variety of techniques^53^, including screen printing, gravure printing, roll-to-roll printing, and inkjet printing. These techniques normally involve screen masks, nozzles, or patterned cylinders for the deposition of the conductive ink at the desired positions and are crucial for defining feature specifications and manufacturing throughput. Furthermore, the translation of these techniques to large-scale roll-to-roll printing offers the possibility for multiarray microsensors to be fabricated over sheets at the meter length scale, thus facilitating large-scale sensor deployment^71^. However, despite their many advantages, thermoplastics are limited in their stretching characteristics and exhibit poor conformability on three-dimensional contours. In comparison, soft elastomeric substrates, which possess similar physical properties to biological skins, are not limited by these issues.

Typically, the fabrication of these soft elastomeric substrates involves a resin mixture and a curing process within a defined mold. Unfortunately, elastomeric substrates normally possess low melting points and high hydrophobicity, complicating fabrication processes based on lithography or inkjet printing. Accordingly, modifications in the fabrication techniques^88–90^, material substitutions^14,91^, and alternative designs^92,93^ have been developed, eventually resulting in flexible soft substrates with improved stretchability and electrical conductivity. Another interesting alternative to all these fabrication techniques is the age-old fabrication process, that is, weaving. This technique requires the implementation of conductive fibers, extruded as thin filaments or used in metalized textile yarns. These fibers can be woven, knitted, or sewn onto textiles to create consumable wearables. Indeed, conductive textiles, now more commonly known as e-textiles, have gained popularity owing to their robustness and versatility. They are capable of sustaining high pressure without mechanical breakage^94^. Currently, conductive textiles have been adopted in various apparel, such as gloves^91,94^, shirts^95,96^, and socks^97^. These particular instances demonstrate the future possibility of integrating flexible sensors into fashionwear for various healthcare and medical engineering applications. Nonetheless, despite its inherent advantages, there still remain technological barriers to the practical implementation of this class of sensors owing to their high cost and low compatibility for electronics integration. Table 1 (C) summarizes some of the processes typically used for the fabrication of flexible physical sensing platforms.

## Solid-state physical sensing platforms

Tremendous advancements have been achieved in recent years in engineering solid-state devices and sensors with a high degree of mechanical deformability and conformability for a multitude of applications. Stretchable and wearable solid-state sensors are typically constructed from highly conductive, elastic, and lightweight materials. They also incorporate solid-state components that function as their active sensing elements. To date, a majority of these solid-state sensing elements are made of various polymer-, carbon-, and metallic conductor-based nanomaterials, such as polymer nanofibers^23,24,32^, silver (Ag) and gold (Au) nanoparticles and nanowires^3,37,98^, CNTs^29–31^, and graphene^81–83,99^. Solid-state nanomaterials have been deemed a suitable candidate for flexible conductors because they possess unique physical properties, including a high aspect ratio, superior electrical conductivity and mechanical strength, and low density. Alternatively, in addition to individual solid-state components, the hybrids or composites of these solid constituents are also being increasingly explored as the sensing elements of flexible and stretchable sensors. One common instance of these hybrid structures is elastomeric composites incorporating conductive nanofillers, with their highly percolating networks serving as the conduction path^98–100^.

**Figure 3:**
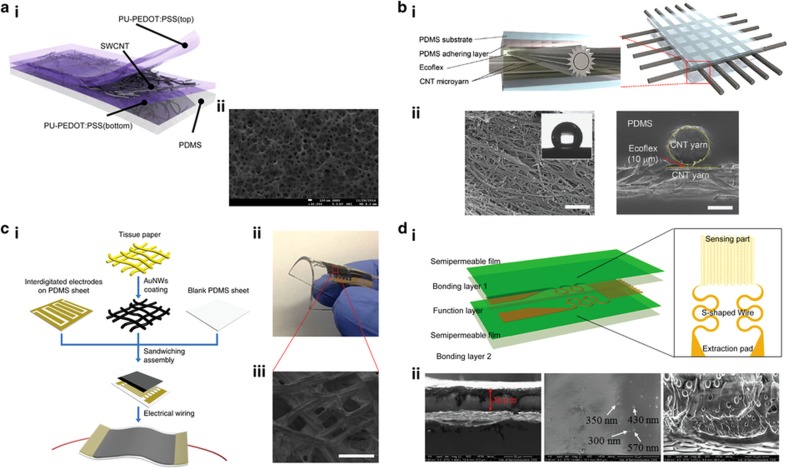
Solid-state physical sensing platforms. (**a**) Stretchable strain sensors based on the nanohybrid assembly of SWCNTs and PEDOT:PSS. (i) Schematic cross-section illustration of the device comprising stacked layers of PU-PEDOT:PSS, SWCNT, and PEDOT:PSS on a PDMS elastomer. (ii) Scanning electron microscope (SEM) image showing the top view of the three-layer nanohybrid strain sensor. Adapted with permission from Ref. 34. Copyright 2015 American Chemical Society. (**b**) All-carbon multimodal piezocapacitive stretchable skin sensor. (i) Schematic illustration showing the active layers of the hierarchically engineered CNT microyarn-based sensor. (ii) SEM image of the surface of the CNT microyarns (left). Scale bar, 1 μm. Inset shows the hydrophobic nature of the surface. Cross-sectional SEM image of the CNT microyarn-incorporated layered structure (right). Scale bar, 50 μm. Adapted with permission from Ref. 35. Copyright 2015 Wiley-VCH Verlag GmbH & Co. (**c**) AuNW-coated tissue paper-based flexible pressure sensor. (i) Schematic illustration showing the fabrication process of the pressure sensor. (ii) Optical image showing the flexibility of the fabricated device. (iii) SEM image showing the surface morphology of the AuNW-coated tissue paper. Scale bar 100 μm. Adapted with permission from Ref. 3. Copyright 2014 Macmillan Publishers Limited. (**d**) Stretchable and breathable skin-inspired temperature sensor. (i) Device architecture of the temperature sensor. (ii) SEM image showing the cross-section of the semipermeable PU film (left), the surface of the semipermeable PU film (center), and the microstructure of the PU layer of the semipermeable membrane. Adapted with permission from Ref. 101. Copyright 2015 Macmillan Publishers Limited. AuNW, Au nanowire; CNT, carbon nanotubes; PU, polyurethane; SEM, scanning electron microscope; SWCNTs, single-walled CNTs.

In one of the latest studies, Roh *et al.*^34^ described the use of a nanohybrid assembly of single-walled CNTs (SWCNTs) and a conductive composite elastomer comprising poly(3,4-ethyl-enedioxythiophene-poly(styrenesulfonate)) (PEDOT:PSS) and a polyurethane (PU) dispersion for the development of stretchable strain sensors with high sensitivity, reliability, and tunability (Figure 3a). The stretchable strain sensors are attached to different facial and body parts and are capable of detecting and monitoring the skin strains and muscle movements during facial expressions and daily activities. Structurally, the strain sensor is a three-layer stacked structure of PU-PEDOT:PSS/SWCNT/PU-PEDOT:PSS on a PDMS substrate (Figure 3a(i)). In addition, the surface morphology of the SWCNT-based nanohybrid sensor is porous because of the interaction between the CNTs and the top and bottom conductive PU-PEDOT:PSS elastomeric layers (Figure 3a(ii)).

In a separate study, Kim *et al.*^35^ reported the development of highly sensitive multimodal all-carbon skin sensors based on elastic sand highly conductive CNT microyarns (Figure 3b). The solid-state wearable sensor is capable of simultaneously sensing multiple external physical stimuli, including tactile, humidity, temperature, and biological variables. CNT microyarns are generally an assembly of hierarchically engineered CNTs in microscopic 1D fabrics with better mechanical, electrical, and thermal properties than individual CNTs. Architecturally, the multi-stimuli-responsive sensory system employs a piezocapacitive-type device assembly comprising CNT microyarn circuitry and a stretchable elastomer dielectric on PDMS substrates (Figure 3b(i)). In this arrangement, the CNT microyarns were aligned such that there were point-to-point overlaps to obtain a reliable sensor array with high spatial resolution and sensitivity. The surface of the CNT microyarns exhibited a hierarchically structured network of fibers, which contributed to its hydrophobic nature, as well as excellent fatigue or damage resistance under an applied stress (Figure 3b(ii)). These properties enabled the high stretchability and reliability of the assembled device. In fact, the CNT microyarn-based skin sensor displayed superior mechanical robustness, as it maintained its device integrity after being subjected to different mechanical deformations.

Interestingly, Gong *et al.*^3^ reported a wearable pressure sensor developed through sandwiching an Au nanowire (AuNW)-impregnated tissue paper between a blank PDMS sheet and a PDMS substrate patterned with interdigitated arrays of electrodes. The fabrication process of this AuNW-based solid-state pressure sensor is illustrated in Figure 3c(i). Ultrathin AuNWs with extremely high aspect ratio were first synthesized and deposited onto tissue paper through the process of dip-coating and drying (Figure 3c(iii)). The mechanically robust yet flexible AuNW-impregnated tissue paper was then inserted between the two layers of blank and patterned PDMS substrates. As a result of the flexibility of both the AuNWs and the tissue paper, the as-fabricated pressure sensing device is wearable and highly bendable (Figure 3c(ii)).

In another recent example, Chen *et al.*^101^ demonstrated soft, stretchable, and breathable skin-inspired biocompatible temperature sensors based on the integration of a porous semipermeable PU film as the substrate and a temperature-sensitive patterned Au film as the sensing component (Figure 3d). Structurally, the solid-state temperature sensor consisted of five different layers of semipermeable film: the encapsulating layer; bonding layer; functional sensing layer; semipermeable film substrate; and adhesive layer stacked together to form a functional unit (Figure 3d(i)). The functional Au-sensing layer, in particular, consisted of three temperature sensor components, ‘S’-shaped interconnects, and extraction pads. The semipermeable PU films, on the other hand, were porous (Figure 3d (ii)) with pore size larger than air and water vapor molecules but smaller than liquid water droplets and bacteria. As such, the fabricated temperature sensor was waterproof with outstanding air and water vapor permeability while being impermeable to water and bacteria.

Altogether, all these representative works have demonstrated that solid-state flexible and stretchable physical sensing platforms may utilize a wide spectrum of solid-state materials as their active sensing elements. At the same time, the physical properties of these active components dictate the operation mechanisms of the solid-state sensors and, ultimately, the distinct applications they may be suited for.

## Liquid-state physical sensing platforms

In addition to the solid-state flexible sensors, another unique group of flexible sensing platforms have emerged over the last several years with conductive fluid as the active sensing element, that is, liquid-state flexible sensing devices. Generally, liquid-state sensors adopt a microfluidics-based device configuration to confine conductive liquids within soft elastomeric substrate-based templates. Microfluidics-based sensor technology has seen rapid progress in an array of applications, particularly in numerous chemical and biological assays, owing to the unique advantages it offers, such as high device sensitivity and adaptability, minute sample quantity, low power requirements, and low fabrication costs^19,102,103^. Employing only a small volume of working fluid, an external load may be detected and quantified based on the variations in the electrical parameters of the device. These parameter changes are typically driven by the displacement of the conductive fluid in the microfluidic channel owing to changes in the microchannel geometry (that is, length and cross-sectional area) upon load application. Microfluidics-enabled devices, as such, provide an ideal platform for the exploration and development of functional liquid-state devices.

As an alternative to solid-state sensors, liquid-state devices may be, in principle, more attractive and robust for flexible and wearable sensing applications, as liquids are fundamentally more deformable than solids. More importantly, liquids represent the ultimate limit in mechanical deformability. On the basis of intrinsic deformability, liquid-state device technology is anticipated to overcome the limitations of typical solid-state materials, such as plastic deformation, delamination, and fracture. In considering the active liquid-sensing component, working fluids such as ionic and metallic liquids are highly advantageous because they exhibit high conductivity and excellent physicochemical and environmental stability.

**Figure 4:**
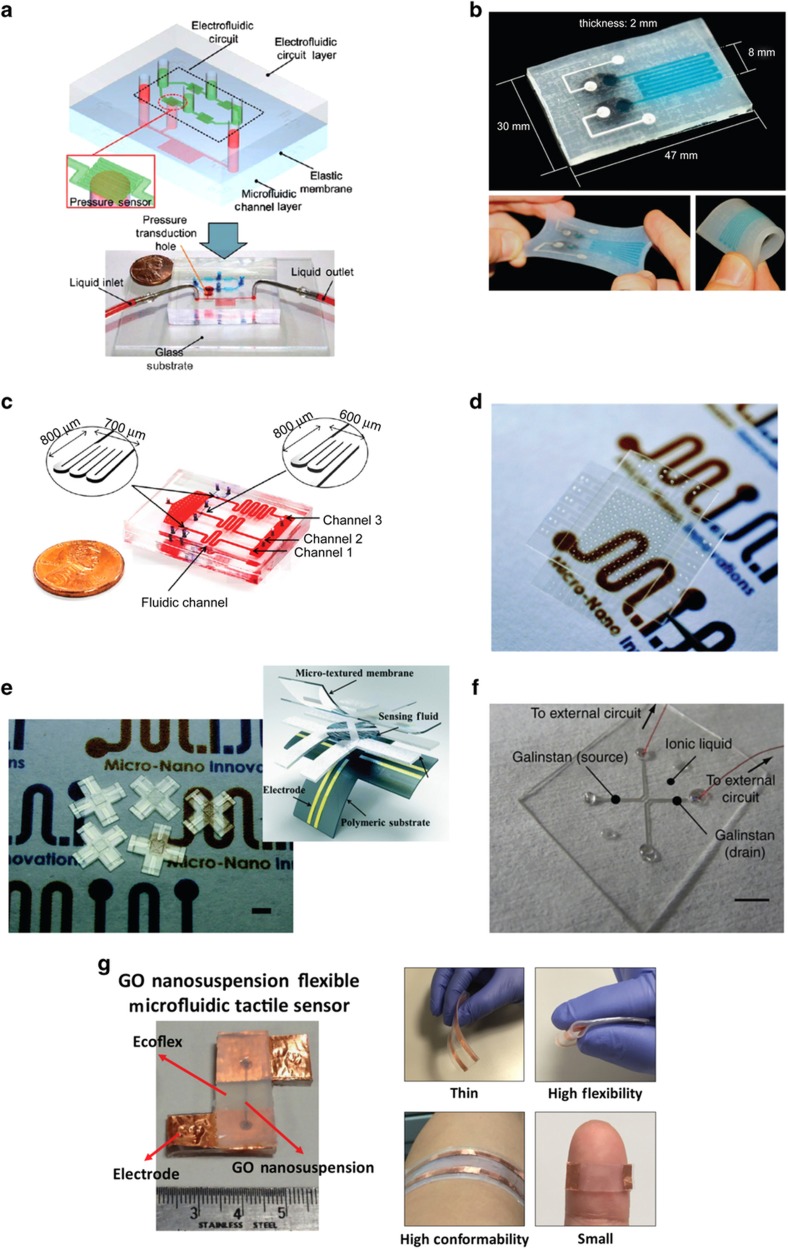
Liquid-state physical sensing platforms. (**a**) Ionic liquid-based electrofluidic pressure sensor. Schematic illustration depicting the device architecture of the pressure sensor and optical image showing the as-fabricated device. The top electrofluidic circuit and the bottom microfluidic channel were filled with blue and red dyes, respectively. Adapted with permission from Ref. 41. Copyright 2011 The Royal Society of Chemistry. (**b**) Hybrid soft strain sensor. Optical image showing the as-fabricated sensor with its stretchability and bendability. Adapted with permission from Ref. 42. Copyright 2013 IEEE. (**c**) Metallic liquid-based microfluidic pressure sensor. Optical image showing the as-fabricated microfluidic pressure sensor with its channel features and dimensions. Adapted with permission from Ref. 43. Copyright 2015 MDPI AG. (**d**) Iontronic microdroplet array (IMA) flexible tactile sensor. Optical image illustrating the fully fabricated IMA tactile sensor array consisting of 12×12 elements. Adapted with permission from Ref. 105. Copyright 2014 The Royal Society of Chemistry. (**e**) Microfluidics-based three-dimensional tactile force sensor. Optical image showing the actual fabricated microfluidic tactile sensing devices for three-dimensional force measurements. Scale bar 2 mm. Inset shows the device architecture of the microfluidics-based three-dimensional tactile force sensor. Adapted with permission from Ref. 106. Copyright 2014 The Royal Society of Chemistry. (**f**) Liquid-state heterojunction sensor. Optical image depicting the actual fabricated liquid-state heterojunction sensor. Scale bar 2.5 mm. Adapted with permission from Ref. 102. Copyright 2014 Macmillan Publishers Limited. (**g**) Graphene oxide (GO) nanosuspension liquid-state microfluidic tactile sensing device. Optical image showing the fully fabricated liquid-state tactile sensor with its distinctive features. Adapted with permission from Ref. 107. Copyright 2016 Wiley-VCH Verlag GmbH & Co.

In an earlier study, Wu *et al.*^41^ demonstrated a liquid-state pressure sensing scheme based on electrofluidic circuits fabricated from ionic liquid-filled microfluidic channels (Figure 4a). The integrated microfluidic device consisted of two PDMS layers patterned with microchannels, that is, a bottom microfluidic channel layer and a top electrofluidic circuit layer, sandwiched by a thin PDMS membrane. The ionic liquid filling the electrofluidic circuit was 1-ethyl-3-methylimidazolium dicyanamide, which is electrically conductive and thermally stable, resulting in the long-term stability of the device. On the basis of this microfluidic setup, pressure sensing could be achieved by measuring the electrical resistance change of the electrofluidic circuit owing to the deformation of the cross-sectional area of the electrofluidic channel upon pressure application. For stable and accurate electrical signal measurements, an electrofluidic Wheatstone bridge circuit was incorporated into the top electrofluidic layer.

In addition to ionic liquids, metallic liquids have been increasingly explored as the active sensing component of flexible microfluidics-based sensing platforms. One of the most commonly used liquid metals for this purpose is the conductive eutectic gallium indium (eGaIn). Possessing a liquid state under room temperature, the metallic alloy of eGaIn has a high conductivity, similar to copper^104^. It is also a non-toxic alternative to mercury. Several studies have shown that the electrically conductive, low-viscosity liquid metallic alloy eGaIn can be patterned as electrodes and configured for pressure sensing and can robustly withstand a high degree of strain. In fact, stretchable sensors based on eGaIn have been demonstrated to be capable of accurate and reliable measurement of large strains with a linear output profile, making them attractive for soft robotics applications. Utilizing two conductive fluids with different resistivity, Chossat *et al.*^42^ developed a soft single-strain sensing element for the measurement of strain triggered by a prosthetic hand’s movements (Figure 4b). In their work, the ionic liquid of NaCl in glycerol solution, which has higher resistivity, was used as the active sensing element in the strain-sensitive part of the sensor. The metallic liquid eGaIn, with lower resistivity, meanwhile, served as the soft wires routing the electrical signal to external electrical circuits. With this arrangement, the soft sensor was highly specific and sensitive only to the prosthetic hand-induced strains while remaining largely insensitive to strains generated elsewhere.

In addition to eGaIn, Galinstan is another commonly used metallic alloy for liquid-state flexible and stretchable physical sensing platform. Galinstan is generally a eutectic alloy of gallium, indium, and tin. Like eGaIn, Galinstan exists in liquid phase at room temperature and possesses high conductivity. In a recent work, Jung *et al.*^43^ demonstrated a Galinstan-based resistive pressure sensor embedded in a microfluidic system (Figure 4c). Interestingly, the as-fabricated liquid metal-based pressure sensor possessed three microfluidic channels for the concurrent acquisition of fluid viscosity under three distinct shear-rate conditions. Much as in the work by Wu *et al.*, the Galinstan-based pressure sensing microfluidic setup was assembled from three layers, that is, the bottom layer consisting of the microfluidic channel, the middle layer consisting of thin PDMS membrane, and the top layer consisting of the pressure sensing channel, and shared the same operating mechanism. Specifically, pressure could be estimated from changes in the electrical resistance of Galinstan owing to the deformation of the cross-sectional area of the top microfluidic pressure sensing layer.

As an emerging microfluidics-based technology, droplet microfluidics have also been explored as a potential candidate for flexible liquid-state physical sensors. In one of the earliest implementations, a highly sensitive mercury droplet-based sensor was demonstrated by sandwiching the droplet between two planar electrodes insulated with a material with high dielectric permittivity. In fact, the advent of droplet microfluidics, coupled with renewed interest in using iontronic materials such as ionic liquids for electronic transport regulation, has propelled the development of novel flexible sensing platforms in the last few years. For example, more recently, Nie *et al.*^105^ presented an iontronic microdroplet array (IMA) device for flexible tactile sensing based on the droplet-enabled interfacial capacitive sensing mechanism (Figure 4d). Structurally, each sensing component of the IMA sensor consists of a nanoliter ionic droplet nestled between two layers of flexible polymeric membranes patterned with transparent electrodes. This configuration results in an electrical double-layer interface with a high unit-area capacitance upon electrolyte–electrode contact, leading to a unique sensing scheme with high sensitivity and fine resolution. The ionic liquid 1-ethyl-3-methylimidazolium tricyanomethanide, with high conductivity, low viscosity, and excellent electrochemical and environmental stability, was utilized as the sensing fluid of the iontronic droplet sensor.

Intriguingly, based on the same interfacial capacitive sensing principle and using the same ionic liquid as the working fluid, Nie *et al.*^106^ demonstrated a microfluidic-based tactile sensor for three-dimensional force measurement (Figure 4e). The microfluidic tactile sensor comprised three microfabricated functional layers, that is, a bottom layer of conductive electrodes patterned on polymeric substrate, a middle layer of microfluidic sensing chambers filled with conductive ionic liquid and detection channels, and a top layer of deformable micro-textured sensing membrane (Inset of Figure 4e). By integrating the common and differential microfluidic sensing components with a topologically micro-textured membrane, the group showed that the device was capable of measuring and resolving both the normal mechanical loads in the *z* axis and the shear loads tangent to the surface in the *x*–*y* axes based on the uniform and differential membrane surface deformations, respectively. These deformations, in turn, affected the interfacial capacitance of each unit of the sensing device. The magnitude and direction of the mechanical loads could be estimated from the corresponding changes in the interfacial capacitance.

Despite advances in liquid-state physical sensing platforms, challenges still remain in the pursuit of fully functional liquid-state sensors and devices. Most liquid-based devices are still limited to a single liquid element, either ionic or metallic liquid, owing to the difficulty in the fabrication of liquid-based junctions, particularly the problem of liquid intermixing. Nevertheless, quantum advancement towards the realization of functional liquid-state systems has been reported recently. In one of the latest efforts, Ota *et al.*^102^ demonstrated a liquid–liquid heterojunction microfluidic device utilizing both metallic and ionic liquids (Figure 4f). In this configuration, instead of the conventional continuous junction, the two liquids were connected through a series of heterojunction microfluidic channels. These junction channels prevented the metallic and ionic liquids from interpenetrating by introducing regions of high flow resistance at the interface between the two liquids. Through the use of different ionic liquids as the sensing element, the liquid–liquid heterojunction microfluidic sensor was responsive to a multitude of stimuli, such as temperature, humidity, and oxygen, with high sensitivity. At the same time, it possessed outstanding mechanical deformability owing to the inherent nature of the liquids. In fact, the heterojunction device exhibited superior mechanical stability and integrity, maintaining its junction interface structure and liquid confinement after being subjected to various forms of deformations, including bending, twisting, grasping, and tying.

Utilizing a unique graphene oxide (GO) nanosuspension, our group recently demonstrated a liquid-based microfluidic tactile sensor (Figure 4g)^107^. Architecturally, our device consisted of two layers of Ecoflex-PDMS soft template sandwiching the working liquid GO nanosuspension. In general, GO, a hydrophilic derivative of two-dimensional graphene, has been actively explored for a myriad of biomedical and biological applications in recent years^108,109^. With high resistivity^110,111^, low differential conductivity^112^, and low surface tension^113^, GO nanosuspension offers high sensitivity as the active sensing element of the resistive tactile sensor. Interestingly, with this device architecture, our wearable sensing platform was highly flexible, deformable, and capable of withstanding various mechanical deformations, such as pressing, stretching, and bending. This example further demonstrated the special features and potential utility of the liquid-state sensor technology.

## Mechanical deformation-based sensing mechanisms

Flexible and wearable solid-state and liquid-state physical sensors and devices normally detect the desired physical data based on force-triggered changes in their specific electrical parameters, such as piezoelectricity, triboelectricity, capacitance^105,106,114^, and resistance^3^. The parameter variations of these active sensing components are largely driven by the mechanical deformations experienced by the devices, such as pressing, stretching, bending, and twisting. These deformations change the cross-sectional area of the device, which subsequently leads to a change in the physical distance between the active sensing element and conductive electrodes of the sensors. In fact, this sensing mechanism has been exploited and adopted in most existing solid-state and liquid-state flexible and wearable physical sensors.

**Figure 5:**
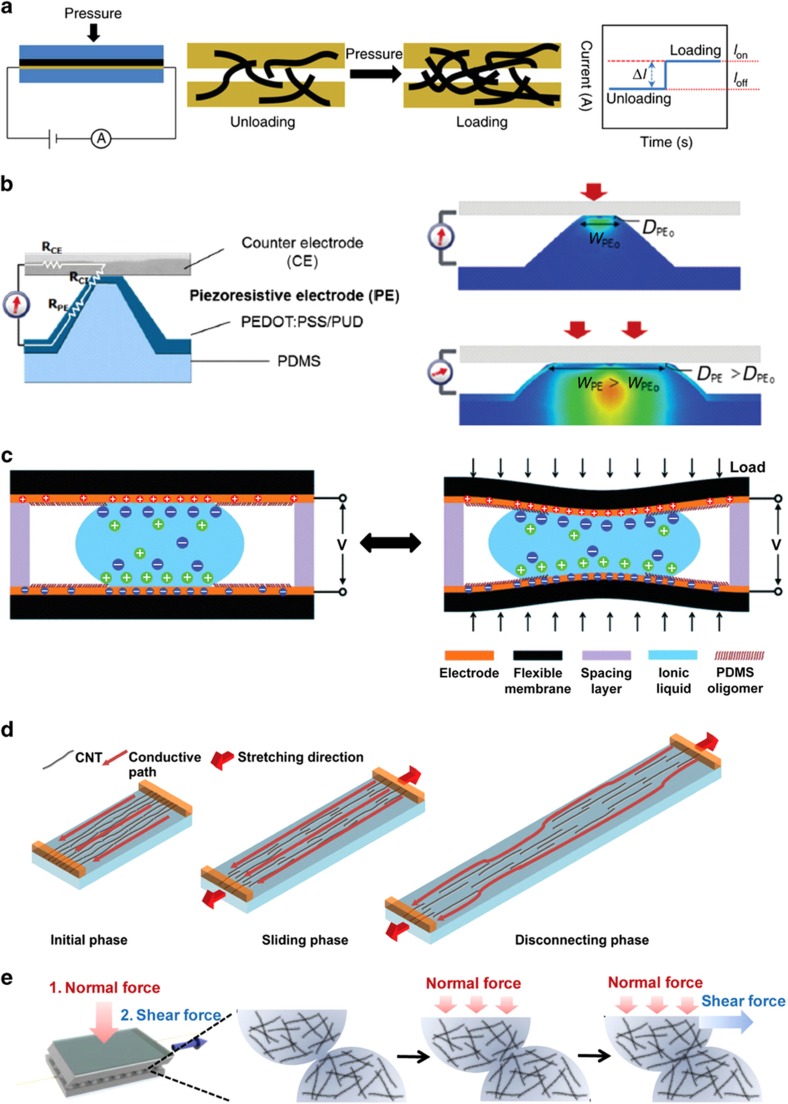
Deformation-based physical sensing mechanisms. (**a**) AuNW-coated tissue paper-based pressure sensor. Schematic illustration depicting the pressure-induced deformation-based working mechanism of the sensor. Adapted with permission from Ref. 3. Copyright 2014 Macmillan Publishers Limited. (**b**) Micropyramid-based stretchable resistive pressure sensor. Schematic illustration showing the circuit model describing the pressure-induced deformation-based sensing principle of the device and the finite element analysis illustrating the distribution of stress on the micropyramid-based electrode upon the application of external pressure. Adapted with permission from Ref. 17. Copyright 2014 Wiley-VCH Verlag GmbH & Co. (**c**) Iontronic microdroplet array (IMA) flexible tactile sensor. Schematic illustration showing the interfacial capacitive sensing principle of the IMA flexible tactile sensor. Adapted with permission from Ref. 105. Copyright 2014 The Royal Society of Chemistry. (**d**) CNT-based elastic strain sensor. Schematic illustration showing the operating principle of the CNT fiber-based strain sensor under different strain regimes. Adapted with permission from Ref. 70. Copyright 2015 American Chemical Society. (**e**) Skin-inspired interlocked microdome array-based tactile sensor. Schematic illustration showing the normal and shear force detection capability of the interlocked microdome arrays based on the distinct surface deformation of the microdomes upon the application of different forces. Adapted with permission from Ref. 7. Copyright 2015 American Chemical Society.

For example, in the work of Gong *et al.*^3^, the developed AuNW-based flexible pressure sensor operated based on the contact between the AuNWs and the interdigitated electrode arrays induced by a pressing force (Figure 5a). The AuNWs were coated on a soft tissue paper, leading to an assembly with rough, porous surfaces filled with interlocking AuNWs. The number of AuNW–electrode pairs contributing to the changes in the electrical parameters of the sensor varied according to the external pressure applied. More specifically, upon the application of an external force, the AuNW-coated tissue paper underwent a compressive deformation, which led to an increase in the amount of AuNWs bridging the finger electrodes, resulting in a higher number of conductive pathways and an increased sensor current. Upon unloading, the reverse occurred. Owing to the recovery of the tissue paper to its original shape, the amount of AuNWs in contact with the electrodes decreased, reducing the sensor current.

The same contact–noncontact pressure sensing mechanism was further explored in the latest work by Choong *et al.*^17^. In that study, the group presented a highly stretchable resistive pressure sensor based on arrays of a micropyramid-patterned elastomer (Figure 5b). The micropyramid elastomer with a spring-like compressible platform was first replicated from a silicon mold and subsequently grafted with the polymer-based stretchable electrode. The conductive electrode comprised an elastomeric blend of conductive polymer PEDOT:PSS and an aqueous PU dispersion. This structural configuration served as a piezoresistive electrode in which the external pressure applied was a function of the electrical resistance change of the sensor. The introduction of a counter electrode in contact with the piezoresistive electrode under external force completed the sensor assembly. By bridging the two electrode terminals, a voltage difference with respect to the piezoresistive electrode would induce the flow of electrical current. In this arrangement, the eventual resistance around the pyramidal structure depended on the total resistances of the piezoresistive electrode, the counter electrode, and the contact interface. When a small pressure was exerted on the device, the counter electrode came into contact with the pyramidal peak. This contact led to the formation of a highly resistive electrical path (high *R*_o_) owing to the small contact perimeter (low *W*_PEo_) and the thin composite polymer electrode coating (low *D*_PEo_). As the contact pressure increased, the micropyramid deformed laterally, resulting in a wider electrode interface (high *W*_PE_) and thicker electrical current path (high *D*_PE_). As such, the device displayed increased current conduction (low *R*). Importantly, by exploiting the pressure-triggered geometrical change of the sensor and the very small shape factor of the micropyramids (that is, the ratio of the compressed area (the pyramidal tip) to the total unloaded surface areas (the triangular walls of the pyramid)), the device was capable of low-pressure sensing with enhanced sensitivity.

Similarly to solid-state flexible sensing platforms, platforms utilizing liquids as the active sensing element may also acquire the desired physical data based on the force-induced variations in the electrical parameters of the sensors. For example, the IMA-sensing device developed by Nie *et al.*^105^ operated based on an interfacial capacitive sensing mechanism in which the sensor capacitance corresponding to an applied external load varied according to the mechanical deformation experienced by the membrane layers sandwiching the ionic liquid (Figure 5c). With the presented device configuration, an electrical double layer (EDL) with a high interfacial capacitance would be established upon direct ionic droplet-electrode contact. Under external loads (for example, intentionally applied load and/or conformal deformation experienced by the device), the flexible membrane surface would be deformed, leading to a circumferential expansion of the interfacial contact between the ionic droplet and the electrode. This area expansion resulted in a corresponding increase in the EDL capacitance, which could be electronically detected.

In addition to the pressure-induced physical sensing principle, the last several years have seen the development of highly flexible and elastic strain sensors in which variations in the electrical properties of these sensors are caused by strain force. Among the plethora of materials being considered, nanoscale carbon materials such as CNTs, with their unique capability of forming conductive networks, have been actively explored as individual strain sensing elements with high elasticity or as conductive fillers within soft polymers for detecting large strains. Ryu *et al.*^70^, for example, fabricated a highly elastic wearable strain sensor for detecting human motion using CNT fibers (Figure 5d). The magnitude of the applied strain force would be detected as a function of the resistance change of the entire device. Instead of the total electrical resistance of individual CNTs, the device resistance depended on the effective contact area between individual CNTs, as these CNTs possessed large contact resistance with each other. In this work, the highly oriented arrays of CNT fibers were attached directly on an elastic silicone elastomer substrate. With this setup, when the device was subjected to a stretching force, a uniform stress would be distributed over the whole assembly, and the stress concentration would be simultaneously reduced. During the initial sliding phase under the low-strain regime between 0 and 200%, an increase in the electrical resistance of the device was observed owing to a decrease in the effective contact area between the sliding CNTs. As the device was stretched further beyond its sliding limit, the CNT fibers might be disconnected. Furthermore, as the physical distance between the disconnected CNT fibers increased, the number of conductive paths decreased, whereas the number of CNT fibers forming conductive paths increased. Eventually, during the disconnecting phase under the high-strain regime beyond 200%, owing to the synergistic effect between the sliding and disconnecting CNT fibers, an increase in the value of the gauge factor was noted.

In addition to individual pressure- and stretch-driven electrical parameter variations, there have been increasing efforts to develop flexible sensing devices with simultaneous and multiple physical data-acquisition capabilities. For instance, Park *et al.* demonstrated the biomimetic design of skin-like interlocked microdome arrays with exceptional tactile sensing capability (Figure 5e)^7^. In fact, changes to the electrical properties of the piezoresistive device were dependent on various stretch, normal, and shear forces. Here the CNT-based composite elastomer films were first microstructured with arrays of hexagonal microdomes. Through the contact and engagement of two microdome-patterned sides, interlocked geometry was then achieved. In the interlocked microdome-based system, the surface deformation patterns of the microdomes upon the application of normal and shear forces were uniquely different owing to the different directions of the mechanical stresses. These differences produced distinct changes in the contact resistance of the microdomes in response to the normal and shear forces. Consequently, the device was capable of detecting and differentiating the magnitude and direction of these two types of loads via their distinct sensory output patterns.

## Emerging healthcare and biomedical applications

### Artificial electronic skins

Biological skin-based sensory receptors (for example, mechano- and thermoreceptors) gather and transmit rich streams of physical variables from the external environment^8^. Despite significant developments in the understanding of mechano- and thermosensations, the replication of these unique sensory capabilities in artificial skins and prosthetics largely remains an elusive goal. Consequently, instead of serving as a functional substitute for natural limbs, prostheses and artificial skins are frequently worn merely as supplementary movement aids^115^ or for cosmetic utility. Recent advances in the design of sensor-laden prosthetics and artificial skins integrated with rigid and semi-flexible sensing devices offer promising alternatives, albeit with limited spatiotemporal resolution, stretchability, and conformability^116^. Moreover, severe mechanical mismatches between the electronics of these biomedical devices and soft biological tissues further impede the utility and performance of these systems.

One of the most actively explored applications of flexible and wearable sensing platforms in healthcare and biomedical applications has been in the development and realization of human-adaptive artificial electronic skins (e-skins) that mimic the spatiotemporal sensing and transduction abilities of biological skins^7,8,44–46^. In the last few years, stretchable e-skins with high sensitivity have garnered tremendous interest because they are capable of emulating human skin functionality in detecting subtle changes in external stimuli, such as pressure, strain, shear, temperature, vibration, and pain, and transducing these data as electronic signals. At the same time, they are highly conformable to soft, curved, and complex surfaces. These properties translate into the potential utility of flexible e-skin in applications such as wearable real-time health monitoring, prosthetic limbs, and rehabilitation devices^117^.

**Figure 6:**
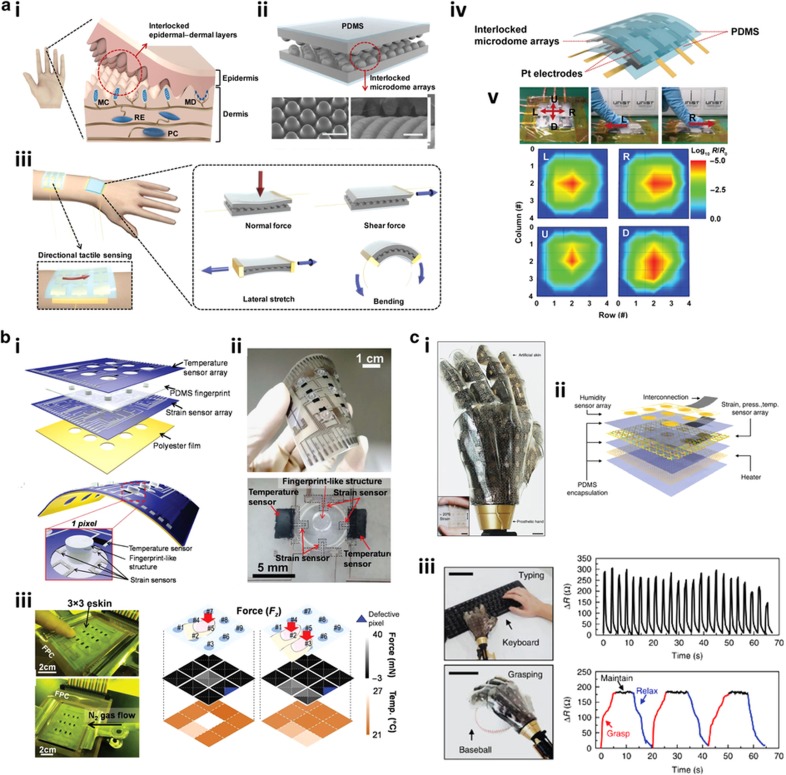
Flexible and stretchable physical sensing platforms for artificial electronic skins. (**a**) Stretchable e-skin configured from CNT-PDMS composite film patterned with interlocked microdome arrays. (i) Schematic illustration of the structure of human skin depicting the interlocked epidermal–dermal layers and the various skin mechanoreceptors. (ii) Schematic illustration of the design of the interlocked microdome arrays and the corresponding tilted and cross-sectional SEM images of the arrays of microdomes on a composite film. Scale bars, 5 μm. (iii) Schematic illustration showing the attachment of the stress-direction-sensitive e-skin on a human arm for the directional tactile sensing and differentiation of a range of mechanical stimuli, such as normal, shear, lateral stretch, and bending forces. (iv) Schematic illustration showing the configuration of 3×3 pixel e-skin arrays sandwiched between the cross-arrays of electrodes and PDMS layers for the three-axial directional sensing of mechanical stimuli. (v) Spatial distribution and directional mappings of external finger pushes applied on the e-skin. Adapted with permission from Ref. 7. Copyright 2015 American Chemical Society. (**b**) Strain-engineered artificial e-skin sensor arrays integrated with a fingerprint-like structure. (i) Schematic illustration showing an exploded view of the device configuration with the corresponding enlarged view of the fingerprint-like structure with its four strain and one temperature sensors. (ii) Optical image showing the actual fabricated e-skin device in a 3×3 array (top) and the corresponding enlarged image of the actual fabricated fingerprint-like structure (bottom). (iii) Optical image and schematic illustrations depicting the two-dimensional force and temperature mapping capability of the 3×3 array e-skin device in response to external stimuli, such as finger touch. Scale bars, 2 cm. Adapted with permission from Ref. 46. Copyright 2015 American Chemical Society. (**c**) Smart prosthetic e-skin sensor constructed from stretchable silicone nanoribbon (SiNR) electronics. (i) Optical image illustrating the smart artificial e-skin with its stretchable SiNR electronics laminated compliantly onto a prosthetic hand. Inset shows a 20% stretched e-skin. Scale bars, 1 cm. (ii) Schematic illustration depicting the exploded view of the device architecture of the smart e-skin. (iii) Optical images of the e-skin-laminated prosthetic hand tapping a keyboard and grasping a baseball and the corresponding temporal resistance changes of the artificial e-skin in response to different external stimuli as captured and monitored by the pressure sensor. Adapted with permission from Ref. 8. Copyright 2014 Macmillan Publishers Limited.

Artificial flexible and stretchable e-skins have been developed from a broad range of micro/nanomaterials and structures with diverse detection modes. For instance, in the work of Park *et al.*^7^ highlighted previously, the design of the interlocked microdome arrays, mimicking human skin epidermal–dermal ridges, was fabricated from CNT composite elastomeric films, and the device operated based on the piezoresistive sensing principle (Figures 6a(i) and (ii)). The conductive CNT-PDMS composite films were initially patterned with arrays of microdomes. Two layers of microdome-structured elastomeric films were then engaged and interlocked to complete the e-skin configuration (Figure 6a(ii)). When attached to human skin, the interlocked microdome array-based e-skins were capable of highly sensitive differentiation of a broad range of applied mechanical stimuli, including normal, shear, stretch, bend, and torsion forces (Figure 6a(iii)). In addition to multiple physical stimuli, the highly stretchable e-skins possessed multidirectional force detection capability. Interestingly, the group further developed e-skins with 3×3 pixel arrays nestled between the platinum electrode arrays (Figure 6a(iv)) to show the applicability of their wearable e-skins in resolving the spatial distributions and directions of various external stimuli (Figure 6a(v)). In fact, the e-skin was able to resolve two different touch positions on its pixels with high sensitivity based on its characteristic spatial mapping of those positions. Furthermore, the device was capable of providing different signal patterns and distinct spatially resolvable mappings according to the magnitude and direction of the applied stimuli, such as finger touches, air flows, and vibrational stimuli. All of these abilities highlighted the three-axial tactile detection capability and the stress-direction sensitivity of the stretchable e-skins with their unique configuration of interlocked microdome arrays.

In another recent study on three-directional multifunctional flexible sensing, Harada *et al.*^46^ demonstrated strain-engineered fingerprint-like sensor arrays for artificial e-skin applications (Figure 6b). The device was capable of detecting three-axis tactile and slip/friction forces as well as temperature distribution. In fact, the changes in the electrical resistance of the device were functions of both force and temperature. Using the screen printing method, the highly integrated device was fully printed on a flexible substrate. Architecturally, the device consisted of a 3×3 array of fingerprint-like structures sandwiched between the arrays of temperature and strain sensors, with the polyester film serving as the base material of the sensing device (Figure 6b(i)). Each pixel of the device comprised four strain sensors for the concurrent detection of the direction of three-axis forces, as well as a temperature sensor, for a total of 36 strain and nine temperature sensors integrated on the flexible substrate. The as-fabricated three-axis force and temperature sensors were mechanically flexible (Figure 6b(ii)). To demonstrate the operational functionality as well as the high sensitivity and specificity of the e-skin device towards force and temperature sensing, the group subjected the 3×3 array artificial skin to human finger touch, finger slip/friction, and N_2_ gas flow (Figure 6b(iii)). On the basis of the distinct force and temperature mappings, the spatiotemporal distributions of the friction force and temperature corresponding to air flow distribution could be monitored and distinguished, demonstrating an e-skin fully functional in mimicking human skin.

In addition to the acquisition of basic physical variables (for example, force and temperature), artificial e-skins equipped with additional functionalities, such as humidity sensing for skin moisture sensation, thermal heating for body temperature regulation, and the ability to interface with the peripheral nervous system, are highly desirable for a more complete system resembling biological human skin. Interestingly, various recent developments in the applications of e-skins are inching towards these goals. One of these advances was demonstrated by Kim *et al.*^8^ in their recent studies in which a stretchable smart prosthetic skin based on ultrathin silicon nanoribbons (SiNRs) was developed for pressure, strain, and temperature sensing (Figure 6c). In addition to the multimodal sensing capability, the tactile and thermal sensor arrays were instrumented with humidity sensors, electroresistive thermal heaters, and stretchable arrays of electrodes for nerve stimulation. The artificial skin with its integrated electronics was highly stretchable, compliant, and able to mechanically couple to the curved surfaces of prosthetics (Figure 6c(i)). Structurally, the e-skin consisted of stacked layers of electronics, sensors, and actuators (Figure 6c(ii)). Specifically, filamentary electroresistive heaters bonded to an elastomeric substrate formed the bottom layer of the prosthetic skin. The middle layer of the e-skin consisted of arrays of pressure, strain, and temperature sensors. The top layer was embedded with humidity sensor arrays. The different sensor and actuator layers were separated from each other by thin elastomeric encapsulating layers and were also individually interconnected to the external data-acquisition devices.

The group subsequently demonstrated the applicability of the artificial e-skin laminated on a prosthetic hand through a series of complex operations, including handshaking, keyboard tapping, ball grasping, cold/hot drink holding, and wet/dry surface touching (Figure 6c(iii)). Intriguingly, minute shifts in strain in the vicinity of the index finger and the respective joints during handshaking could be spatiotemporally mapped by the SiNR strain sensor arrays. Furthermore, the temporal resistance and temperature changes of the device in response to external stimuli such as keyboard tapping, ball catching, and cold/hot drink touching could be rapidly and reliably captured and monitored by the corresponding pressure and temperature sensors. In addition, the SiNR-based humidity sensor of the prosthetic e-skin was capable of highly sensitive sensing of fluid contact-triggered dampness by providing appropriate feedback on the degree of humidity and wetness of the contact surface. Clearly, the collection of mechanically stretchable and durable sensors integrated within the prosthetic e-skins significantly enhanced their localized sensory perceptions and spatiotemporal sensitivity in response to the highly varied stimuli from the external environment.

### Physiological monitoring and assessment systems

Spatiotemporal monitoring and assessment of the physiological properties of soft biological tissues and organs are critically relevant in clinical diagnosis and prognosis. For example, time-dependent changes of the physical properties of a wide range of physiological conditions are central to the clinical monitoring and measurement as these variations are anticipated as a result of alterations in pathophysiology or responses to therapy. The spatial resolution of the screening and examination assays, in particular, is significantly important, as it may reveal certain physiological microscale disorders^47^.

**Figure 7:**
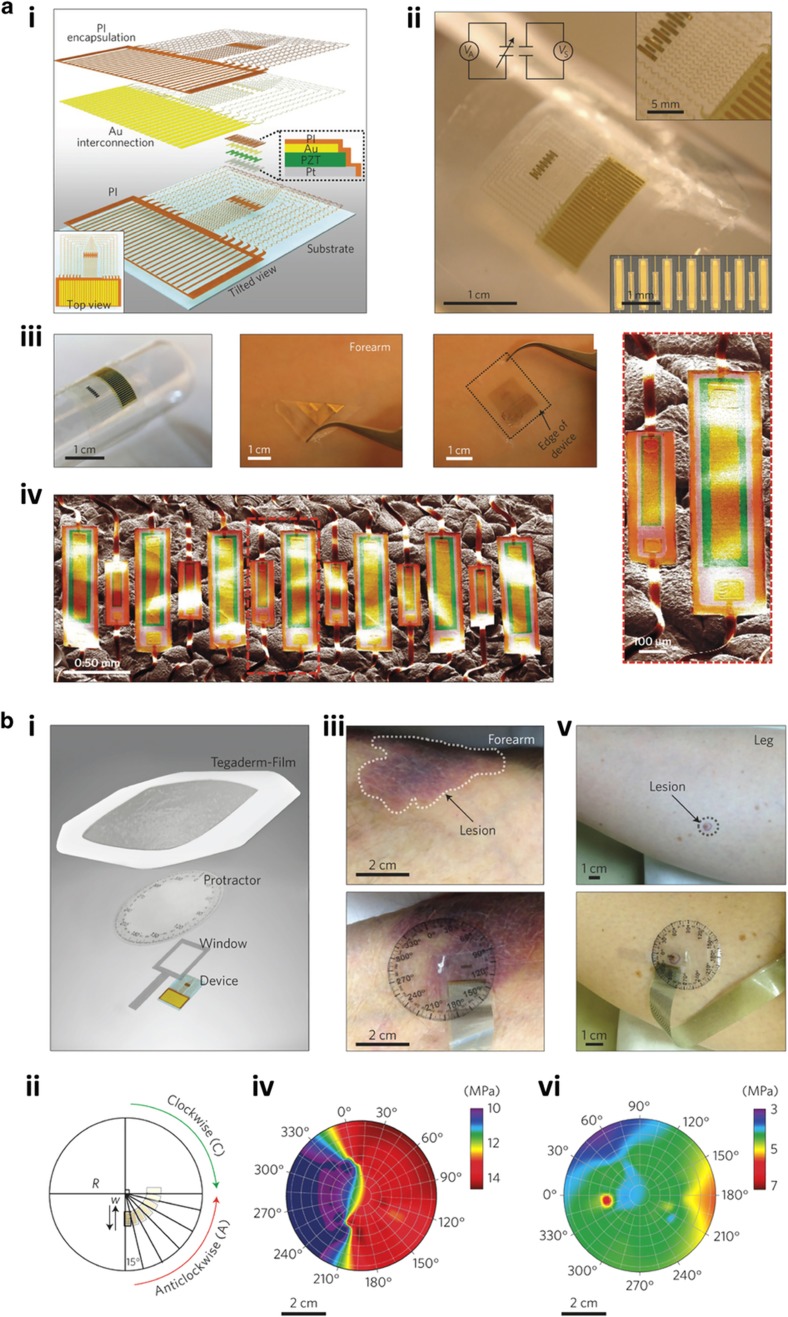
Flexible and stretchable physical sensing platforms for physiological monitoring and assessment. (**a**) Soft piezoelectric compliant modulus sensor (CMS) constructed from the flexible networks of mechanical sensors and actuators based on lead zirconate titanate nanoribbons. (i) Schematic illustration showing the exploded view of the device architecture: the top view of the device is shown in the lower-left inset, whereas the cross-sectional view of the device is depicted in the black-dashed region. (ii) Optical image showing the actual fabricated device on a thin silicone substrate. Scale bar 1 cm. Insets show the gold interconnection region (upper right, scale bar, 5 mm) and the arrays of sensors and actuators (lower-right, scale bar 1 mm) with the corresponding electrical circuit diagram (upper left). (iii) Optical images of a device conformed onto a cylindrical glass (left) and a device laminated partially (center) and fully (right) on the skin. Scale bars 1 cm. (iv) SEM image showing a CMS unit consisting of an array of six sensors and seven actuators on an artificial skin (scale bar 0.5 mm) with the corresponding magnified image of the red-dashed region depicting a sensor (left) and actuator (right) pair (scale bar, 100 μm). (**b**) Spatiodirectional mapping capability of the rotatable CMS unit and *in vivo* ‘on patient’ assessment. (i) Schematic illustration showing the exploded view of the rotatable CMS unit. (ii) Diagram illustrating the spatiodirectional mapping principle of the device, where *R* defines the protractor radius, and w defines the distance between the protractor center and the first sensor edge in the array that describes the mapping region. (iii) Optical image showing the forearm in the absence (top) and presence (bottom) of the mounted device. (iv) Mapping data corresponding to the assessment in (iii). (v) Optical image showing the lower leg in the absence (top) and presence (bottom) of the mounted device. (vi) Mapping data corresponding to the assessment in (v). Adapted with permission from Ref. 47. Copyright 2015 Macmillan Publishers Limited.

One of the emerging potential applications of flexible and conformal sensing platforms is in the spatiotemporal characterization and evaluation of the fundamental physical properties of organs such as skin, with high resolution under either static and/or dynamic conditions. In one of the most recently described major breakthroughs, Dagdeviren *et al.*^47^ devised ultrathin, soft conformal piezoelectric devices capable of *in vivo* characterization of the mechanical properties of soft tissues in the near-surface sections of the epidermis (Figure 7). The devices were constructed from flexible networks of mechanical sensors and actuators using lead zirconate titanate nanoribbons, and they conformed to the underlying complex surface textures of the skin and other organs under both static and dynamic conditions (Figure 7a). Importantly, these reversible laminated systems on the soft tissues enabled rapid and non-invasive quantitative evaluation of viscoelasticity, coupled with the spatial mapping functionality. The active conformal modulus sensor (CMS) with serpentine-shaped connecting metal traces had an ultrathin configuration and yielded a low modulus with stretchable mechanics when integrated with a thin elastomer (Figure 7a(i)). On the basis of the van der Waals forces alone, the stretchable device could be simply and non-invasively attached onto the skin surface and other soft biological tissues (Figure 7a(ii and iii)). Interestingly, the measurement accuracy of the devices was minimally compromised by multiple applications and removals from the soft tissues (Figure 7a(iii)). In general, a CMS unit comprised an array of rectangular structures of six sensors and seven actuators (Figure 7a(ii and iv)). These structures are capacitive components in which the top and bottom electrodes are separated by a piezoelectric material.

By exploiting the reversibility of the contact between the CMS unit and the soft tissues, the piezoelectric device was able to measure and provide spatial and directional mappings of the regional stiffness (Figure 7b). As a demonstration, the modulus sensor was aligned to the window element and integrated with a transparent film-printed protractor equipped with a rotatable part (Figure 7b(i)). Using this architecture, the device could be calibrated in either the clockwise or anticlockwise direction (Figure 7b(ii)). To prevent undesirable movements during data recording, an adhesive film was applied to the whole sensing structure. As a proof-of-concept of the ‘on patient’ measurements, the configured device was attached onto the skin of a basal cell carcinoma cancer patient, particularly at the lesion sites on the forearm (Figure 7b(iii)) and leg (Figure 7b(v)). The spatial and directional mappings of the associated measurements on the forearm (Figure 7b(iv)) and leg (Figure 7b(vi)) were subsequently obtained. Overall, the potential utility of stretchable conformal sensors for rapid ‘on patient’ detection and spatiotemporal mapping of the physical properties of soft tissues and other vital organs has been suitably demonstrated.

Importantly, with the rapid progress in the design and implementation of flexible and wearable sensing platforms for physiological monitoring and assessment, it is anticipated that exciting advances in the development of devices with better architectural design and features, such as further miniaturized device configuration, increased spatial resolution, and enhanced sensitivity, will be realized in the near future for this particular application in healthcare and medical engineering.

### Therapeutic and drug-delivery platforms

**Figure 8:**
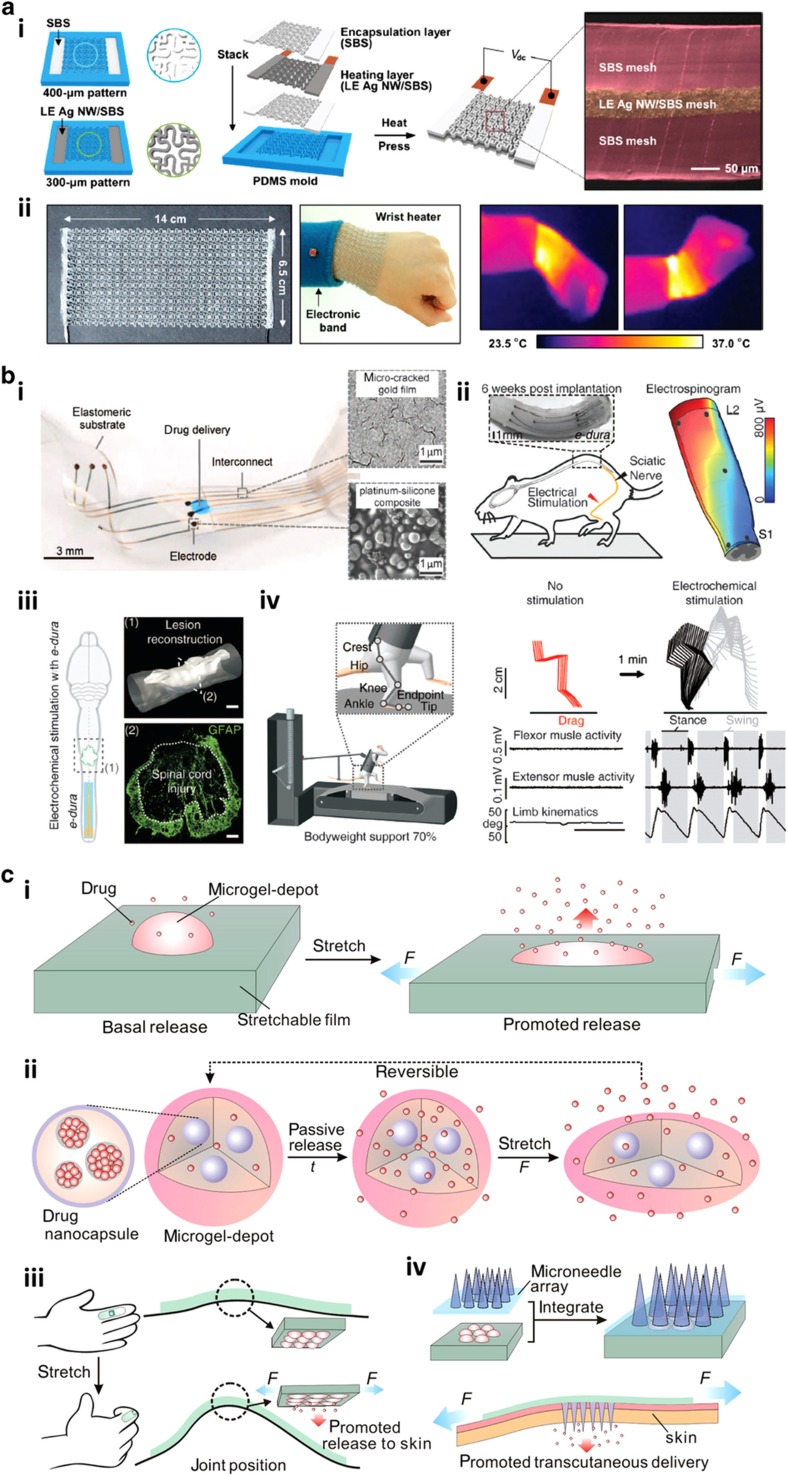
Flexible and stretchable physical sensing platforms for thermal therapy and drug delivery. (**a**) Stretchable and conformal mesh heating element for articular thermotherapy application. (i) Schematic illustration showing the fabrication process of the stretchable mesh heater, which comprised a heating layer of LE Ag NW/SBS elastomer composite and two encapsulation layers of SBS elastomers pressed together at high temperature. The colorized SEM image on the right shows the good interface between the three bonded mesh layers of SBS, LE Ag NW/SBS, and SBS. Scale bar, 50 μm. (ii) Optical image showing the large-area stretchable mesh heater (left). Optical image showing a wearable and portable heating system that integrated the stretchable mesh heater and a custom-made electronic band and the application of the integrated heating system on a wrist (center). Infrared camera images showing uniform heat distribution on the wrist (right). Adapted with permission from Ref. 48. Copyright 2015 American Chemical Society. (**b**) Soft elastic electronic dura mater or e-dura neural implants. (i) Optical image illustrating the fabricated e-dura implant and the accompanying SEM images of the stretchable gold interconnects and platinum–silicone composite-coated soft electrodes. (ii) Implantation of the e-dura between the motor cortex tissues and the dura mater for 6 weeks (left) and the reconstructed spinal cord activation map in response to electrical stimulation of the left sciatic nerve based on the recorded electrospinograms (right). (iii) Spinal cord injured rats with implanted spinal e-dura over the lumbosacral sections. (iv) Recording of the bipedal locomotion of the rat under support after 3 weeks of rehabilitation in the absence and presence of electrochemical stimulation and corresponding stick diagram decompositions of the hindlimb movements and oscillations and the leg muscle activities. Adapted with permission from Ref. 49. Copyright 2015 American Association for the Advancement of Science. (**c**) Wearable tensile strain-triggered drug delivery system. (i) Schematic illustration showing the two distinct components and the working mechanism of a strain-triggered drug delivery system in which deformation of the stretchable elastomer promoted drug release from the microdepot. (ii) Schematic illustration showing the encapsulation of the drug-loaded nanoparticles within the microdepot and the passive release and partial retention of the drug-filled nanoparticles within the microdepot matrices. (iii) Conformal attachment of the wearable drug delivery system onto the index finger where drug release to the skin could be simply triggered by the finger flexion. (iv) Integration of the wearable strain-responsive drug delivery system with a microneedle array patch for the transcutaneous administration of drugs. Adapted with permission from Ref. 50. Copyright 2015 American Chemical Society.

In addition to exciting applications such as artificial e-skins and physiological monitoring and assessment systems, another emerging use of soft flexible and wearable physical sensing platforms is for therapeutic and drug delivery purposes^48–50^. An interesting example is a recent study published by Choi *et al.*^48^ presenting the fabrication of a stretchable and conformal heating element composed of a nanocomposite of Ag nanowires and thermoplastic elastomer for articular thermal therapeutic application (Figure 8a). In general, thermal therapy is one of the physiotherapy techniques used in orthopedics to treat joint injuries and alleviate the associated symptoms. Conventional thermal therapy utilizes heat packs and wraps for continuous point-of-care heat treatments to maximize the therapeutic effects^118,119^. Nevertheless, technical drawbacks, such as the difficulty of temperature control coupled with wearability issues, including mechanical rigidity, bulkiness, and heaviness, which cause discomfort to the wearers, limit the uses of the current therapeutic methods. As such, Choi *et al.* explored the development of a stretchable and wearable heating element that was thin and lightweight in a portable form.

First, the formation of a highly conductive and homogenous nanocomposite comprising Ag nanowires and styrene-butadiene-styrene (SBS) elastomer was achieved through a ligand exchange (LE) reaction. The LE Ag NW/SBS elastomer nanocomposite was subsequently patterned in a full serpentine mesh configuration using prefabricated molds to maximize the system’s softness and stretchability (Figure 8a(i)), resulting in device conformability on moveable curvilinear joints as well as effective heat conduction. The nanocomposite-based heating layer was then sandwiched between the two insulating SBS encapsulation layers and pressed together at high temperature to form the final serpentine mesh heater. A wearable and portable heating system for continuous point-of-care articular thermotherapy was engineered by integrating the large-area stretchable mesh heater with an electronic band equipped with a battery for the power supply (Figure 8a(ii)). Interestingly, the entire soft mesh heater generated heat uniformly and stably owing to the homogeneity and high conductivity of the conductive elastomer and simultaneously maintained its conformal contact with the skin despite large joint flexions and muscle extensions.

In one of the noteworthy breakthroughs reported recently, Minev *et al.* engineered soft elastic electronic neural interfaces with long-term biointegration and functionality in the central nervous system (Figure 8b)^49^. The introduced neural implants, called electronic dura mater or e-dura, mimicked the shape and mechanical property of the dura mater, that is, the membrane protecting the brain and spinal cord. In general, advances in the modulation and recording of neural activities have spurred the development of a myriad of implantable neuroprostheses. Nevertheless, to date, the long-term biointegration and the anticipated therapeutic benefits of these neural implants have not materialized owing to the significant biomechanical mismatch between the stiff implantable neuroprostheses and the soft neural tissues^120,121^. Consequently, Minev *et al.* exploited soft neurotechnology in tailoring their e-dura implants. Architecturally, the e-dura integrated an optically transparent silicone elastomeric substrate, platinum–silicone composite-coated soft electrodes, stretchable gold interconnects, and a compliant microfluidic structure (Figure 8b(i)). The elastomeric substrate enabled simultaneous optical stimulation and neural recording. The electrical excitation pulses and electrophysiological signals were transmitted using the platinum–silicone composite electrodes and gold interconnects. The microfluidic structure served as a chemotrode for localized chemical drug delivery. The e-dura implant displayed an outstanding stretchability owing to the use of soft electrodes and the presence of microcracks in the interconnects.

The e-dura, consisting of a 3×3 electrode array, was inserted between the motor cortex tissues and the dura mater for the electrocorticograms, whole-body kinematics, and leg muscle activity recording of freely behaving rats. The electrospinograms in response to the motor cortex or the sciatic nerve stimulation were also measured to verify the neural recording capability of a chronically implanted e-dura over spinal tissues (Figure 8b(ii)). Motor command and peripheral sensory feedback were reliably detected and recorded with distinct spatiotemporal selectivity. The e-dura was eventually evaluated for locomotion restoration after a paralyzing injury to the spinal cord. First, the adult rats were clinically subjected to permanent paralysis of both legs by inflicting a contusion at the thoracic level, leaving <10% of the spinal tissues at the lesion epicenter (Figure 8b(iii)). Next, the e-dura was used to engage the spinal locomotor circuits in the vicinity of the injury site. A serotonergic replacement therapy was then delivered through the microfluidic-based chemotrode, while electrical stimulation was concurrently transmitted. Remarkably, through the simultaneous and colocalized electrochemical stimulations, the paralyzed rats were eventually able to walk (Figure 8b(iv)). The e-dura clearly facilitated reliable neural therapy throughout the 6-week rehabilitation. On the whole, the elastic and durable e-dura neural implants with integrated modalities afforded numerous neuroprosthetic applications. Specifically, they were capable of high-resolution neuronal recordings and delivered concurrent electrochemical spinal neuromodulation for the restoration of locomotion after a paralyzing spinal cord injury while simultaneously inducing minimal foreign-body reaction and reduced side effects in the subdural space.

Di *et al.*^50^, on the other hand, recently introduced a wearable and tensile strain-triggered drug delivery system integrating a stretchable elastomer and microgel depots consisting of drug-loaded nanoparticles (Figure 8c). Stimulus-induced drug delivery systems may enable a dose- and spatiotemporally controlled sustained release of therapeutics^122,123^. Nonetheless, the clinical translation of these systems is still challenging because stimuli relying on physiological changes are usually limited by the precise dose control in the physiological environment^124^, whereas the external factor-driven techniques are limited by the need for complex instrumentation. Mechanical strain-associated stimuli are seen as a viable alternative, as they are generally capable of providing a simple and accessible method of spatiotemporally controlled drug delivery. Furthermore, the strain-triggered drug delivery scheme is promising for the self-administered release of either analgesic or emergency drugs based on simple body movements. Such body-motion-induced strain variations may be achieved through tensions in muscles, tendons, and bone joints, compression in bones and cartilage, or shear in blood vessels^125^. Nevertheless, till now, there are still few strain-triggered drug delivery systems available, and a majority of the existing ones are based on compression, that is, compression-controlled drug release. The low strain tolerance of these systems greatly limits their use in applications with a high degree of deformation. Thus, the realization of stretchable drug delivery systems with high drug-loading capacity as well as large deformation capability for long-term therapeutic applications remains elusive.

The tensile strain-triggered therapeutics delivery system developed by Di *et al.*, in contrast, demonstrated that both simple body motion-triggered sustained release and intentionally administered pulsatile release of drugs could be efficiently achieved. Structurally, the introduced drug delivery system consisted of two distinct components: crosslinked microgels encapsulating drug-filled polymeric nanoparticles, which served as microdepots for the sustained release of drugs, and stretchable elastomer, which served as the base substrate for tensile strain loading (Figure 8c(i)). The drugs loaded in the nanoparticles were passively released and partially retained in the microdepot matrices (Figure 8c(ii)). Owing to an increased surface area for diffusion coupled with compression on the microdepots, the drug release from the microdepots would be initiated as soon as a tensile strain was applied (Figure 8c(iii)). The stretch-responsive system was further integrated with polymer-based microneedle arrays for transcutaneous insulin delivery and the eventual regulation of the blood glucose level of chemically induced diabetic mice (Figure 8c(iv)). This work showed that the application of tensile strain to a stretchable device via daily body motions or deliberate stretching facilitated the effective and controlled release of therapeutics. Crucially, by integrating the system with microneedle arrays, the transcutaneous administration of small drugs, hormones, or vaccines to the body for different disease treatments could be achieved.

All of these examples have highlighted the numerous potential applications of flexible, stretchable, and wearable physical sensing devices as an enabling technology for therapeutic and drug delivery. It is anticipated that, with the integration of other wearable modalities, fully functional and complete drug delivery and therapeutic platforms capable of monitoring real-time physiological signals and providing appropriate feedback to guide the precise application of specific therapy or drug delivery can be effectively realized.

## Challenges and opportunities

In this review, we have provided a state-of-the-art overview of the emerging flexible physical sensing platforms for healthcare and biomedical applications. As highlighted here, the development of flexible and wearable physical sensing devices is among the emerging technological goals for a wide spectrum of applications, especially those related to healthcare and medicine. For these particular applications, the sensing platforms should be designed and tailored such that their sensing parameters, particularly sensitivity and range of detection, are within the ranges required for distinguishing different bodily movements and/or extracting other physiological information. For instance, physical sensors targeted for applications associated with standing and sitting motions need to account for the pressures exerted by these actions, which range widely from 10 to 500 kPa (Ref. 126). In fact, these ranges of pressures are unique to different bodily actions. For example, the pressure exerted when one is in a supine position is between 2 and 5 kPa. In addition, a gentle finger touch, wrist pulse, and intraocular pressure may range from 1 to 10 kPa (Refs. 127,128), 0.2 to 3 kPa (Refs. 128,129), and 1 to 4 kPa (Ref. 130), respectively. Consequently, to meet the stringent demands of these healthcare and medical applications, highly sensitive sensing platforms with a typical sensitivity ranging from ~10 kPa^−1^ down to 0.001 kPa^−1^ or a gauge factor between 0.005 and 100 (Table 2) are generally required. In addition, these physical devices are required to conform to soft and complex surfaces with high curvilinear features, such as biological skins. As a result, flexible sensing devices constructed from different materials (that is, both solids and liquids), equipped with a variety of deformation-based sensing mechanisms (that is, press, shear, stretch, and bend), and able to transmit the detected physical information through variations in different electrical parameters (that is, resistance, capacitance, piezoelectricity, and triboelectricity) have been investigated and explored in recent years.

Undoubtedly, the rapid progress in the development of flexible sensors and devices in the last several years has highlighted the growing importance and potential utility of this special class of sensing platforms. Promising advances in these sensing technologies are evident, and different levels of maturity in terms of device operation and implementation have been demonstrated. Nevertheless, as is the case for most new developments, there are many challenges and opportunities facing this relatively new field, both in the fundamental and applied aspects. These challenges and opportunities include the three areas of design, fabrication, and integration of the developed flexible sensors and devices to enhance the application-specific sensing capabilities, such as sensing modality, functionality, and directionality.

To date, among the studies reported on flexible sensing platforms, a majority have focused mainly on platforms with a single modality in a unidirectional manner, that is, the detection of one specific tactile stimulus in one particular direction, specifically normal pressure or in-plane strain. In fact, there are still limited flexible sensors and devices that operate based on other deformation-triggered sensing principles, such as friction/slip and torsion. Difficulties in device design and fabrication on flexible substrates have been touted as one of the contributing factors. As such, the developed sensors are incapable of detecting and discriminating the types and directions of external stimuli other than the ones they are specifically designed for. In terms of specific applications such as artificial e-skins, this limitation poses a challenge, as artificial e-skins are expected to possess similar capabilities and functionalities to biological skins in terms of spatiotemporal sensing and differentiation of external stimuli to which they are subjected, with the simultaneous transduction of these detected data as electronic signals. Consequently, flexible sensing platforms should be designed and fabricated such that they enable concurrent multimodality and multidirectionality in detecting multiple external stimuli. More importantly, to mimic biological skin more plausibly, the flexible sensors and devices should also integrate other functionalities, such as temperature and humidity sensing, as physiological skin-to-skin contacts entail both tactile stimuli and variations in temperature and humidity.

In addition to the specific application of artificial e-skins, the design and implementation of flexible sensing platforms should also be significantly improved for other applications in healthcare and medical engineering, such as drug delivery and therapeutic applications. In fact, the realization of a wearable and portable stand-alone system is one interesting direction worth pursuing, and we anticipate this goal being achieved through the integration of flexible sensors with other wearable devices. For example, physical sensing platforms (for example, a motion-triggered drug administration system) may be integrated with other wearable modalities to detect and monitor real-time physiological signals, such as body temperature, electrocardiography, or blood glucose level, to provide appropriate feedback to guide specific drug delivery for therapeutic purposes.

Finally, in addition to device design and fabrication, device integration is of utmost importance for the complete realization of stand-alone, multimodal, multifunctional, and multidirectional flexible physical sensing platforms. To implement the developed flexible sensors and devices as fully integrated and functional sensing platforms, all components with different sensing capabilities must be effectively incorporated into a single pixel and subsequently manipulated for large-area integration. At the same time, other factors, such as seamless integration with data processing and wireless transmission systems, are also crucial for bringing the envisioned physical sensing platforms closer to reality. This development may require sophisticated and advanced fabrication processes and therefore necessitate the rational selection and design of sensing materials and device configurations.

Overall, despite all the ongoing challenges for the practical implementation of flexible and wearable physical sensing platforms, there are encouraging positive signs from the growing efforts to address those highlighted areas. Indeed, we foresee and anticipate exciting progress in the near future towards the realization of functional flexible sensing platforms for various healthcare and biomedical applications.

## Figures and Tables

**Table 1:** Summary of the materials and processes typically used for the fabrication of flexible and stretchable physical sensing devices

**A**	Flexible templates	Young’s modulus (MPa)	Tensile strain (%)	Poisson’s ratio	Processing temperatures (^o^C)	Ref.
Polymeric Substrates	Polyethylene terephthalate (PET)	2,000–4,100	<5	0.3–0.45	70	^51–53^
	Polycarbonate (PC)	2,600–3,000	<1	0.37	150	^53^
	Polyurethane (PU)	10–50	>100	0.48–0.49999	80	^54,55^
	Polyethylene naphthalate (PEN)	5,000–5,500	<3	0.3–0.37	120	^52,53,87^
	Polyimide (PI)	2,500–10,000	<5	0.34–0.48	270	^53,86^
Silicone Elastomers	Polydimethylsiloxane (PDMS)	~ 0.36–0.87	>200	0.49999	70–80	^53^
	EcoFlex	~ 0.02–0.25	>300	0.49999	25	^53^
	DragonSkin	1.11	>300	0.49999	25	^56^
	Silbione	~ 0.005	>250	0.49999	25	^56–58^
**B**	**Active sensing elements**	**Structure/form**		**Size**	**Sheet resistance**	**Ref.**
Conductive materials	Metallic nanomaterials (e.g., Ag, Au, Cu, Al, Mn, Zn)	Nanoparticles, nanowires, nanorods		2–400 nm (in diameter) and 200–1000 nm (in length)	0.015–20 Ω sq^−1^	^53^
	Carbon-based nanomaterials (e.g., CNTs, graphene)	Nanoparticles, nanowires, nanotubes, nanofibers		10–2000 nm (in diameter) and 500–5000 nm (in length)	30–5×10^6^ Ω sq^−1^	^53,62–65^
	Ionic or metallic liquids (e.g., eGaIn, Galinstan)	Liquid		Not applicable	2.63×10^−9^–0.025 Ω cm^−1^	^84,85^
**C**	**Fabrication techniques**	**Resolution (μm)**	**Throughput (m min^−1^)**	**Limitations**		**Ref.**
Additive processes	Gravure printing	50–500	8–100	Limited resolution due to alignment		^53^
	Screen printing	30–700	0.6–100	Small selection of inks due to high viscosity requirements Requires hard masks to be replaced regularly		^53^
						
	Inkjet printing	15–100	0.02–5	Not suitable for roll-to-roll production Coffee-ring effect Limited printing area		^53^

**Table 2:** Summary of the sensitivity, resolution, and detection limit of some of the demonstrated flexible physical sensing platforms

Sensing platforms	Sensor types	Sensitivity	Unit	Resolution	Unit	Detection limit	Unit	References
Solid-state	AuNW-based pressure sensor	>1.14	kPa^−1^	N.A.		13	Pa	3
Solid-state	CNT-based tactile sensor	27.8 to 9617	G.F.	N.A.		100	Pa	7
Solid-state	SiNR tactile sensor	0.000315 to 0.0041	kPa^−1^	N.A.		87	kPa	8
Solid-state	PEDOT:PSS-based pressure sensor	4.88 to 10.3	kPa^−1^	N.A.		0.37 to 5.9	kPa	17
Solid-state	ITO-based pressure sensor	8.4×10^-5^ to 0.45	kPa^−1^	1 to 5	mm	1 to 1800	kPa	19
Solid-state	CNT-based tactile sensor	0.004	G.F.	N.A.		50	kPa	29
Solid-state	CNT-based strain gauge	1	G.F.	N.A.		N.A.		30
Solid-state	CNT-based strain sensor	0.06 to 0.82	G.F.	N.A.		N.A.		31
Solid-state	Pt nanofiber-based strain gauge	0.75 to 11.45	G.F.	N.A.		5	Pa	32
Solid-state	CNT/PEDOT:PSS-based strain sensor	62.3 to 109	G.F.	N.A.		N.A.		34
Solid-state	CNT-based tactile sensor	0.034 to 0.05 (for <0.1 kPa) and 0.5 (for >10 kPa)	kPa^−1^ (for <0.1 kPa) and MPa^−1^ (for >10 kPa)	N.A.		0.4	Pa	35
Solid-state	AgNW-based pressure sensor	0.88 to 5.54	kPa^−1^	5	Pa	8	Pa	37
Liquid-state	eGaIn-based strain sensor	0.97 to 3.57	G.F.	5%	Strain	5%	Strain	42
Liquid-state	Galinstan-based pressure sensor	N.A.		2.5	kPa	2.5	kPa	43
Solid-state	CNT/PEDOT:PSS/AgNP-based temperature and strain sensor	0.01	mN^−1^	10	μm	1	mN	46
Solid-state	CNT-based strain sensor	0.26 to 1.13	G.F.	N.A.		N.A.		70
Solid-state	Graphene-based strain sensor	2.4 to 14	G.F.	N.A.		N.A.		81
Solid-state	Graphene-based strain sensor	0.11 to 9.49	G.F.	20	μm	N.A.		82
Solid-state	AgNW-based strain sensor	2 to 14	G.F.	N.A.		N.A.		98
Solid-state	Graphene-based strain sensor	10 to 35	G.F.	N.A.		N.A.		99
Solid-state	Carbon black-based strain sensor	1.8 to 5.5	G.F.	N.A.		N.A.		100
Liquid-state	1-ethyl-3-methylimidazolium tricyanomethanide-based tactile sensor	0.43	nF kPa^−1^	1 to 3	mm	33	Pa	105
Liquid-state	1-ethyl-3-methylimidazolium tricyanomethanide-based pressure sensor	29.8	nF kPa^−1^	5	mm	100	mN	106
Liquid-state	GO nanosuspension-based tactile sensor	0.0338	kPa^−1^	N.A.		7	mN	107
Solid-state	Au-based tactile sensor	0.001 to 0.01	kPa^−^^1^	N.A.		5 to 405	kPa	114

Abbreviations: G.F., gauge factor; N.A., not applicable.
